# ASO Author Reflections: In the Era of Neoadjuvant Chemotherapy for Pancreatic Cancer, Should the Definition of Resection Margin Change or Stay the Same?

**DOI:** 10.1245/s10434-025-17460-0

**Published:** 2025-05-13

**Authors:** Won-Gun Yun, Wooil Kwon

**Affiliations:** 1 Department of Surgery and Cancer Research Institute, Seoul National University College of Medicine, Seoul, Republic of Korea; 2 Department of Surgery, Seoul Metropolitan Government-Seoul National University Boramae Medical Center, Seoul National University College of Medicine, Seoul, Republic of Korea

## Past

Over the past few years, the use of neoadjuvant chemotherapy in pancreatic cancer is growing, and more clinical trials have been conducted to assess the efficacy of neoadjuvant chemotherapy even in resectable pancreatic cancer.^1,2^ Although the most crucial aspect of surgery is obtaining a microscopically negative resection margin, the importance of resection margin in the context of neoadjuvant chemotherapy is still poorly understood.^3,4^

## Present

We evaluated the 307 patients with pancreatic head cancer who underwent upfront pancreaticoduodenectomy (PD) and 97 who received neoadjuvant chemotherapy followed by PD. Margin status was divided into a three-tier system: R0-wide (tumor-free margin ≥ 1 mm), R0-narrow (0 mm < margin < 1 mm), and R1 (margin = 0 mm). In the context of upfront surgery, obtaining a wide margin guaranteed better survival outcomes than a narrow margin, however, in the context of neoadjuvant chemotherapy, obtaining a wide margin could not improve survival outcomes compared with a narrow margin.

## Future

These findings indicate that the interpretation of resection margins should be different based on the treatment strategy and extensive surgery to obtain a wide margin could be justified only in the upfront surgery setting.^5^ While these results are straightforward, there are still limitations in accurately assessing margins preoperatively and intraoperatively. To enable surgeons to make comprehensive plans regarding the surgical extent considering an appropriate resection margin, future research will be required on high-resolution imaging techniques that can detect viable tumors prior to surgery and intraoperative pathology confirmation techniques that overcome the inaccuracy of current frozen examinations.
